# The Link between Dietary Protein Intake, Skeletal Muscle Function and Health in Older Adults

**DOI:** 10.3390/healthcare3030529

**Published:** 2015-07-09

**Authors:** Jamie I. Baum, Robert R. Wolfe

**Affiliations:** 1 Department of Food Science, University of Arkansas, 2650 N. Young Ave., Fayetteville, AR 72704, USA; 2 Department of Geriatrics, University of Arkansas for Medical Sciences, 4301 W. Markham St. #806, Little Rock, AR 72705, USA; E-Mail: rwolfe2@uams.edu

**Keywords:** aging, older adults, dietary recommendations, protein, essential amino acids, muscle protein synthesis

## Abstract

Skeletal muscle mass and function are progressively lost with age, a condition referred to as sarcopenia. By the age of 60, many older adults begin to be affected by muscle loss. There is a link between decreased muscle mass and strength and adverse health outcomes such as obesity, diabetes and cardiovascular disease. Data suggest that increasing dietary protein intake at meals may counterbalance muscle loss in older individuals due to the increased availability of amino acids, which stimulate muscle protein synthesis by activating the mammalian target of rapamycin (mTORC1). Increased muscle protein synthesis can lead to increased muscle mass, strength and function over time. This review aims to address the current recommended dietary allowance (RDA) for protein and whether or not this value meets the needs for older adults based upon current scientific evidence. The current RDA for protein is 0.8 g/kg body weight/day. However, literature suggests that consuming protein in amounts greater than the RDA can improve muscle mass, strength and function in older adults.

## 1. Introduction

The older adult population in the United States is a segment of unprecedented growth [1]. Longer life spans and aging of baby boomers will result in doubling of the population of Americans over the age of 65 over the next thirty years to reach almost 90 million people. By 2030, twenty percent of the US population will be comprised of older adults [1]. Chronic diseases such as heart disease, cancer and diabetes pose a great risk as people age. A majority of health care costs for older Americans can be attributed to treatment of chronic diseases [1]. People living with chronic disease(s) often experience diminished quality of life, which can be attributed to a long period of decline and disability [1].

Muscle mass, strength and function are progressively lost with aging [2]. A loss or reduction in skeletal muscle function often leads to increased morbidity and mortality either directly, or indirectly, via the development of secondary diseases such as diabetes, obesity and cardiovascular disease [2,3]. Sarcopenia is the age-associated loss of muscle mass and function. Sarcopenia can appear as early as age 40, increasing significantly over the age of 80, often resulting in 50% or greater loss of muscle strength [3]. The causes of sarcopenia appear to be multifactorial and include poor nutrition, diminished responsiveness to the normal anabolic effect from hormones and/or nutrients, and a sedentary lifestyle. The etiology of sarcopenia includes malnutrition, increased inflammatory cytokine production, oxidative stress, hormone reduction (e.g., growth hormone and testosterone), and decreased physical activity [4,5]. Maintaining skeletal muscle function throughout the lifespan into old age is essential for independent living and good health. The efficient activation of the mechanistic processes that regulate muscle development, growth, regeneration and metabolism is required for skeletal muscle to function at optimal levels [6].

Additional physical changes with aging may occur as a result of changes in skeletal muscle mass [2]. For example, body composition shifts as we age, resulting in a higher percentage of body fat and decreases in muscle mass with age [7]. This imbalance between muscle mass and fat mass occurs even in the absence of changes in body mass index (BMI) [7,8]. The prevalence of obesity among aging adults has also increased over the last several decades. For example, the prevalence of obesity among men aged 65–74 increased from 31.6% in 1999–2002 to 41.5% in 2007–2010. Between 2007 and 2010, approximately 35% of adults age 65 and over were obese [1].

Nutrition is an important modulator of health and function in older adults. Inadequate nutrition can contribute to the development of sarcopenia and obesity [2,5]. As life expectancy continues to rise, it is important to consider optimal nutritional recommendations that will improve health outcomes, quality of life and physical independence in older adults [1].

Several studies identify protein as a key nutrient for older adults (reviewed in [2,5]. Protein intake greater than the amount needed to avoid negative nitrogen balance may prevent sarcopenia [9], help maintain energy balance [10], improve bone health [11,12,13,14], cardiovascular function [15,16,17] and wound healing [18]. Benefits of increased protein intake may improve function and quality of life in healthy older adults, as well as improve the ability older patients to recover from disease and trauma [2].

## 2. Dietary Guidelines for Protein Intake: Current Recommendations May Underestimate Optimal Intake in Older Adults

### 2.1. Current Guidelines for Protein Intake

The most recent Dietary Guidelines for Americans were issued in 2010 by the USDA and were derived from the dietary reference intakes (DRIs) recommended by the Food and Nutrition Board of the Institute of Medicine [19,20]. The DRIs for macronutrients (Table 1) include an estimated average requirement (EAR), a recommended dietary allowance (RDA) and an acceptable macronutrient distribution range (AMDR) [19]. A comparison of the current dietary recommendations for dietary protein intake can be found in Table 1. In the case of daily protein intake, the EAR for dietary protein is 0.66 g/kg/day and the Food and Nutrition Board recommends an RDA of 0.8 g/kg/day for all adults >18 years of age, including older adults >65 years. The RDA for protein was based on all available studies that estimate the minimum protein intake necessary to avoid a progressive loss of lean body mass as determined by nitrogen balance [2,5]. The problem with relying only on results from nitrogen balance studies to define protein intake is that this method does not measure any physiological endpoints relevant to health, a point that was recognized by the Food and Nutrition Board [2]. In addition, the existing data were almost entirely from college-aged men [21]. Translating data from such a single age and gender group becomes an issue when trying to create universal recommendations for all adults over the age of 18, since older adults require greater nitrogen intake to maintain nitrogen balance compared to young adults [5]. Although the Food and Nutrition Board acknowledged this issue [20], they nonetheless relied on these data for the estimation of the RDA and did not differentiate the needs of young adults *versus* older adults [5].

The Food and Nutrition Board recognizes a distinction between the RDA and the level of protein intake needed for optimal health. The recommendation for the ADMRs (Table 1) includes a range of optimal protein intakes in the context of a complete diet (e.g., inclusive of additional macronutrient intake (e.g., carbohydrate and fat)), which makes the ADMR more relevant to normal dietary intake than the RDA [2]. The ADMR recommends that 10%–35% of daily energy intake come from protein [20].

Experts in the field of protein and aging recommend a protein intake between 1.2 and 2.0 g/kg/day or higher for older adults [2,5,22]. Clearly the RDA of 0.8 g/kg is well below these recommendations and reflects a value at the lowest end of the AMDR. Furthermore, current recommendations for protein intake for older adults fail to address needs of clinical conditions such as injury, hospitalization, surgery, *etc.*, which are common in older adults and have been shown to require protein intake above recommended levels.

**Table 1:** Dietary protein intake recommendations.

Recommendation	Gram protein/kg body weight/day
EAR	0.66
RDA	0.8
AMDR *	~1.05–3.67

EAR: Estimated Average Requirement; RDA: Recommended Dietary Allowance; AMDR: Acceptable Macronutrient Distribution Range; * Calculated assuming energy expenditure of 42 kcal/kg/day.

### 2.2. Quantity versus Quality of Protein Intake

It is not only the quantity of protein intake that is important for optimal health in older adults, it is the quality of the protein [23]. There are three important aspects to take into consideration when discussing protein quality: The characteristics of the specific protein, the food matrix in which the protein is consumed, and the characteristics of the individuals consuming the food (e.g., age, health status, physiological status and energy balance) [23]. Current accepted methods for measuring protein quality do not consider the different metabolic roles the essential amino acids (EAA: leucine, valine, isoleucine, lysine, methionine, phenylalanine, tryptophan and histidine) play in the body beyond being the first limiting amino acid for growth or nitrogen balance [23]. Additionally, there are gaps in our knowledge regarding the influence of different protein sources on the metabolic health of older individuals.

Essential amino acids, especially the branched-chain amino acid leucine, are potent stimulators of muscle protein synthesis via the protein kinase mTORC1 (mammalian target of rapamycin complex 1) [24,25,26]. Several studies demonstrate that maximal stimulation of muscle protein synthesis is possible with 15 g of EAA (reviewed in [27]). This translates to ~35 g of high quality protein delivering ~15 g of EAA. A larger amount of lower quality protein, which contains a lower content of EAA, would be required to achieve the same functional benefits. The addition of nonessential amino acids to a supplement containing EAA does not result in additional stimulation of muscle protein synthesis [28], indicating that the quality of the protein, or its amino acid profile, is a key determinant of the functional potential of protein. This is supported by several studies demonstrating that ingestion of milk proteins stimulates muscle protein synthesis to a greater extent after resistance exercise compared to ingestion of soy protein [29,30,31,32]. The data from the Health, Aging and Body Composition study supports these findings [32]. These data show that intake of animal protein, not plant protein, was significantly associated with preservation of lean body mass over three years in older adults [32]. Individuals in the highest quintile of protein intake had 40% less loss in lean body mass than those in the lowest quintile of protein intake [32].

The majority of published results indicating a potential beneficial effect of increasing protein intake in older adults are either from epidemiological or short-term studies. Two recent publications by Tieland *et al.* [33,34] indicate that a significant proportion of the caloric intake above the RDA for caloric intake should be in the form of protein for older individuals. These conclusions are based on the results of randomized, double-blind, placebo-controlled, longer-term (24 weeks) studies. Improved physical performance in frail older adults results was reported when dietary protein intake was increased by twice-daily consumption of 15 g of milk protein. These studies emphasize the importance of choosing the right protein for supplementation. Milk protein was used in these studies [33,34]. The proportion and profile of essential amino acids in milk protein make it a high-quality protein [35]. One important item to note with regard to these studies is that the control group was consuming 1.0 g protein/kg/day, which is 20% higher than the current RDA.

In addition, the difference in digestibility and bioavailability of a protein can impact the quantity of protein that needs to be ingested to meet metabolic needs. The speed of protein digestion and absorption of amino acids from the gut can influence whole body protein anabolism [36]. Proteins with differing amino acid profiles exhibit different digestion and absorption rates [36,37,38,39,40,41,42]. Amino acid availability depends directly on both the quality and quantity of the dietary source of nitrogen [37]. For example, the digestion and absorption rates of fast (e.g., whey) *versus* slow (e.g., casein) proteins need to be taken into consideration when developing protein recommendations. When young, healthy subjects were provided with either a whey protein meal (30 g) or a casein meal (43 g), both containing the same amount of leucine, and whole body protein anabolism was measured, the subjects consuming the whey (fast) protein meal had high, rapid increase in plasma amino acids, while subjects consuming the casein (slow) protein meal had a prolonged plateau of essential amino acids [40]. In addition, consumption of whey protein stimulated postprandial protein synthesis by 68%, while consumption of casein only stimulated protein synthesis by 31%. However, ingestion of casein inhibited whole body protein breakdown by 34%, while whey protein had no effect [40]. These results indicate that slow proteins, when adjusted for leucine content, may promote postprandial protein deposition by an inhibition of protein breakdown. When the influence of protein digestion rate on protein turnover was tested in older subjects, the opposite occurs [39]. When older men consumed 20 g of whey (2.5 g leucine), casein (1.7 g leucine) or casein hydrolysate (1.7 g leucine), the whey protein stimulated postprandial protein accretion more effectively than either casein or casein hydrolysate [39]. These results demonstrate that older and younger individuals respond differently to protein source and dose and highlight the need for separate protein recommendations for young and older adults.

Whether or not the amino acid source is an intact protein or a mixture of free amino acids can also influence the rate muscle protein synthesis [41]. For example, when older subjects were given either an EAA mixture (15 g) or a whey protein supplement (13.6 g) after an overnight fast, subjects consuming the EAA mixture had higher mixed muscle fractional synthetic rate [41]. The differing response could be due to differing leucine content between the supplements (EAA; 2.8 g leucine and Whey; 1.8 g leucine) or because the EAA supplement was composed of individual amino acids while the whey protein supplement was intact protein. This could influence rate of appearance of the amino acids into circulation and protein synthetic response. Another example is the form or texture of the protein itself, such as minced beef *versus* a beefsteak [42]. Older men consumed 135 g as either intrinsically labeled minced beef or beefsteak, which allowed direct assessment of the rate of appearance of dietary protein-derived amino acids from the gut into the circulation. Meat protein-derived phenylalanine appeared more rapidly in the circulation after minced beef than after beefsteak consumption. Whole-body protein balance was higher after consumption of the minced beef *versus* the beefsteak. Muscle protein synthesis did not differ between the two meals over a six-hour period [42]. These data indicate that format of protein during ingestion impacts digestion, absorption and rate of appearance of amino acids into circulation.

The interaction of protein with other substrates (e.g., fats, carbohydrates) may impact functionality of protein, especially at the molecular level. It is well established that the EAA, especially leucine, are essential for the functional benefits associated with increased protein intake in older adults, such as muscle protein synthesis [43,44,45,46]. However, most of these studies have tested either isolated EAA or protein independently of carbohydrates and fat. In the presence of the additional macronutrients, the pool of available EAA could be slower to appear when compared to studies examining EAA, alone, resulting in a decreased muscle protein synthesis response. Animal studies have shown that both fiber and fat can alter gastric emptying rate and delay the rate of appearance of EAA into the blood [47,48]. In healthy older adults, there has been no association with increased protein consumption and detrimental health effects. In contrast, detrimental effects have been associated with excess intake of both fat and carbohydrate. Therefore, not only should the beneficial effects of protein be considered when making dietary recommendations, but also the associated impact of lowering the ratio of fat and carbohydrate in the diet. Several studies have shown that a diet containing 30% protein or greater is beneficial in reducing cholesterol [49], improving glycemic regulation [43,44,50] and improving body composition (e.g., the ratio of lean to fat mass) [51]. These beneficial effects could be due to increasing dietary protein, alone, or a combination of increasing dietary protein and decreasing the amount of carbohydrate, however further investigation is needed.

Older adults are less responsive to low doses of amino acid intake compared to younger individuals [46]. However, this lack of responsiveness in older adults can be overcome with higher levels of amino acid consumption [46]. This is also reflected in studies comparing varying levels of protein consumption [52]. This suggests that the lack of muscle responsiveness to lower doses of amino acids in older adults can be overcome with a higher level of protein intake. The requirement for a larger dose of protein to generate responses in older adults similar to the responses in younger adults provides the support for a beneficial effect of increased protein in older populations [5].

### 2.3. Timing of Protein Intake

Another issue related to defining the optimal protein recommendations for older adults is defining the optimal timing of protein intake throughout the day. Adults typically consume the majority of their protein (and energy) intake at dinner [53], 38 g *versus* 13 g of protein at breakfast. However, ingestion of more than 30 g of protein in a test meal does not further stimulate muscle protein synthesis [54]. Mamerow *et al.* [55] examined the effect of daily protein distribution on skeletal muscle protein synthesis in healthy young men and women for seven days. Ninety grams of protein was either distributed equally or unequally (60% of daily protein intake at dinner) throughout the day. They found even distribution of protein intake throughout day was more effective at stimulating 24 h protein synthesis compared to an uneven distribution [55]. This is further supported by Murphy *et al.* [56] who examined how dietary protein distribution affects muscle protein synthesis in overweight/obese older men under conditions of energy balance, energy restriction and energy restriction with the addition of resistance exercise. Protein was provided as a balanced distribution (25% of daily protein per meal, four times per day) or as a skewed pattern (7%:17%:72%:4% of daily protein per meal) for four weeks. They found that fed-state muscle fractional synthesis rates were higher following the balance protein intake pattern compared to the skewed protein intake pattern under energy balance and energy restricted conditions independent of resistance exercise [56].

A recent study published by Kim *et al.* [57] fails to confirm the importance of spreading protein intake out over the course of the day. Muscle protein synthesis was measured in healthy older adults after consumption of one of four differing daily patterns of protein intake: protein intake at the RDA level (0.8 g/kg/day), protein intake at two times the RDA level (1.5 g/kg/day), either evenly (33% protein at each meal) or unevenly (65% of protein at dinner) distributed throughout the day. In older adults, muscle protein synthesis was greater at two times the RDA than at RDA levels with no significant difference between meal patterns [57]. Likewise, Arnal *et al.* [58], conducted a study with older women fed protein at a dose of 1.7 g/kg fat free mass/day either spread over four meals throughout out the day or in one pulse feeding in which 80% of the daily protein intake was provided at the lunch meal for 14 days. Protein turnover and protein synthesis rates were higher in the pulse fed group compared to when protein was evenly distributed throughout the day [58]. However, in the study by Arnal *et al.* [58], the women only received a total of 65 g/d of protein with the distribution of protein spread throughout four meals with less than 20 g of protein per meal, while in the pulse treatment, the single large meal provided approximately 50 g of protein. This suggests that small meals spread throughout the day may fail to optimally stimulate muscle protein synthesis which could have negative effects on muscle mass. Additional studies compared pulse feeding (72% of daily protein at lunch) *versus* protein being evenly distributed over four daily mails in hospitalized older patients for six weeks [59,60]. These studies found that pulse feeding of protein increased postprandial amino acid bioavailability [59] and increased lean mass [60] compared to spreading protein intake throughout the day. These data may differ from the study conducted by Mamerow *et al.* [55] due to differences in study population (e.g., young *versus* older adults). In addition, the study by Mamerow *et al.* [55] only measured protein synthesis, which is only half of the equation of net protein gain.

## 3. Functional Benefits of Protein in Older Adults

### 3.1. The Role of Leucine in Older Adults

The current RDA for protein may not provide adequate EAA for optimal metabolic roles in adults. It is estimated that 7 to 12 grams of the branched-chain amino acid leucine are necessary per day [44] to see functional benefits beyond nitrogen balance, such as improved muscle function, glycemic regulation and mitochondrial function. Leucine may be more important in older adults than in young individuals. Availability of amino acids following a meal is lower in older adults when compared to young adults; therefore, it takes a greater protein content to elicit the same response postprandial. Several studies have demonstrated that older adults need a greater amount of leucine compared to young adults in order to achieve an increase in muscle protein synthesis [46,61,62].

### 3.2. Increased Muscle Protein Synthesis and Leucine Intake in Older Adults

The mechanism by which dietary protein affects muscle is through the stimulation of muscle protein synthesis by the absorbed amino acids consumed in the diet [27,63]. However, there appears to be an EAA threshold when it comes to stimulating muscle protein synthesis. Ingestion of relatively small amounts of essential amino acids (2.5, 5 or 10 g) appears to increase myofibrillar protein synthesis in a dose-dependent manner [64]. However, larger doses of EAA (20 to 40 g) fail to elicit an additional effect on protein synthesis in young and older subjects. Similar results were observed after ingestion of either 113 or 340 g of lean beef containing 10 or 30 g EAA, respectively [54]. Despite a three-fold increase in EAA content, there was no further increase in protein synthesis in either young or older subjects following consumption of 340 g *versus* 113 g of protein.

It is widely accepted that signaling via mTORC1 (mammalian target of rapamycin complex 1) is involved in the regulation of several anabolic processes including protein synthesis [26,65,66]. In skeletal muscle, amino acids signal through mTORC1 to regulate skeletal muscle protein synthesis [25,67,68,69]. The eukaryotic initiation factor 4E binding protein 1 (4E-BP1) and p70S6K, which play important roles in the initiation of mRNA translation, are downstream targets of mTORC1 [67,68,69]. Signals provided by EAA, especially leucine, are required for full activation of protein synthesis [25,67,70]. With increasing age, muscle may become resistant to the normal stimulatory effects postprandial leucine concentrations [46], which may result in reduced stimulation of the mTORC1 pathway. This could be due to a reduced sensitivity to leucine with age or to the fact that the older adults often have low dietary protein intake [5]. It is estimated that 38% of adult men and 41% of adult women have dietary protein intakes below the RDA. In addition, the decline in the rate of amino acid absorption followed by a decrease in insulin secretion may contribute to the insensitivity of muscle protein synthesis to amino acid-induced stimulation in older individuals [71,72].

Age-related muscle loss may involve a decreased response to EAA due to dysregulation of translation initiation factors. Older adults also have decreased levels of translational proteins related to muscle protein synthesis as compared to young adults [64,73]. Older adults have significantly less skeletal muscle mTORC1 and p70S6K protein levels compared to young adults [64]. In response to 10 g of EAA, mTORC1 phosphorylation, or activation, while significantly increased in skeletal muscle of older adults, is still significantly lower than younger adults. Guillet *et al.* [73] found that p70S6K phosphorylation is not stimulated in older adults after infusion with leucine. These findings are supported by Fry *et al.* [74] who found that older adults have significantly reduced phosphorylation of mTORC1 and translation initiation factors compared to young adults after a bout of resistance exercise. Gene expression of proteins associated with muscle protein synthesis also differs between young and older adults [74]. While no difference was found between young and old in the fasted state, there was a significant decrease in protein (REDD1, TSC1, TSC2 and IGF1 receptor) expression 3 and 6 h post exercise and leucine intervention in older adults *versus* young adults. In addition, after only seven days of bed rest, older adults had a reduced response to EAA ingestion resulting in no increase in muscle protein synthesis, activation of translation initiation factors (4E-BP1 and p70S6K) and no increase in amino acid transporters [75,76]. These findings are striking since pre-bed rest older adults had a significant increase in muscle protein synthesis following ingestion of EAA. These data are important because they demonstrate how quickly a hospital stay could impact skeletal muscle function.

It is also possible that the beneficial effect of protein intake on body composition is due to the stimulation of IGF-1 (insulin-like growth factor 1) secretion. Aging individuals have lower levels of IGF-1 [77], which could contribute to a decrease in protein synthesis rates and accentuate the loss of muscle mass leading to sarcopenia [78]. However, this may be able to be corrected by nutrition intervention since increased protein intake has been shown to increase circulating IGF-1 levels in older adults [79].

## 4. Risk Factors Associated with Low Protein Intake

There are several risk factors associated with reduced protein intake in older adults [22]. These risk factors include reduced energy need and intake, reduced mobility, changes in food preference and food insecurity [22]. For example, as people age, their total daily energy needs decrease and their daily intake of dietary protein progressively declines, with approximately 8% of older women consuming protein below the estimated average requirement [53]. This could be due to underlying disease, changes in appetite and food preference or dental issues [80]. In addition, about 20% of homebound older adults have protein intakes less than the RDA [81], possibly attributed to difficulty acquiring and preparing food due to issues with mobility.

## 5. Clinical and Financial Impact of Nutrition in the Older Adults

It is important to consider optimizing health care and those factors influencing health outcomes when determining dietary recommendations for dietary intake in older adults [2]. The cost of providing health care for one person over the age of 65 is three to five times more costly than for younger adults [1]. This could be because the average length of hospital stay for patients 65 and older is two days longer than younger age groups [1]. Ninety-five percent of health care costs for older Americans are for chronic diseases, which may be attributed, in part, to a loss of functional capacity related to reduced muscle mass [2]. Health care spending will increase by 25% by 2030, primarily due to an increasing older population [1].

Inadequate nutritional intake (e.g., low protein intake) is common in older adults and may explain the depleted muscle mass. Once in the hospital, physical inactivity combined with inadequate protein intake can result in additional loss of muscle mass which can delay recovery and contribute to higher readmission rates [5]. The breakdown of muscle in hospitalized older patients generates the amino acid precursors necessary for synthesis of proteins required for several body processes that are essential for recovery [82]. Many older patients do not have enough muscle mass to endogenously supply the amino acids they need, so the amino acids they need must be provided by dietary protein. However, protein needs are often not met in institutionalized and hospitalized settings [83,84]. There are several benefits associated with increased protein intake in the older individuals, including increased muscle protein synthesis [49,71,85,86] as well as aid in recovery from trauma [87,88] and surgery [79]. Taken together, higher protein intake improves the rate of recovery in older patients, which, in turn, may decrease the length of a hospital stay, indicating that dietary protein can play a central role in patient care [2].

## 6. Conclusions

There is sufficient evidence that protein intake higher than the current dietary recommendations (0.8 g/kg/day) is beneficial for most older adults. Higher protein intakes are associated with increased muscle protein synthesis, which is correlated with increased muscle mass and function. This, in turn, is linked to improved physical function. The evidence presented in this review supports the need for a higher RDA for protein for older adults in order for them to achieve optimal protein intake. Many older people suffering from chronic disease (e.g., diabetes, heart disease) have reduced appetite and do not consume adequate levels protein. Therefore, it is essential that they have adequate protein intake from high quality protein sources.

## References

[1] Centers for Disease Control and Prevention, U.S. Department of Health and Human Services (2013). The State of Aging and Health in America 2013.

[2] Wolfe R.R. (2012). The role of dietary protein in optimizing muscle mass, function and health outcomes in older individuals. Br. J. Nutr..

[3] Arthur S.T., Cooley I.D. (2012). The effect of physiological stimuli on sarcopenia; impact of notch and Wnt signaling on impaired aged skeletal muscle repair. Int. J. Biol. Sci..

[4] Kim T.N., Choi K.M. (2013). Sarcopenia: Definition, epidemiology, and pathophysiology. J. Bone Metab..

[5] Wolfe R.R., Miller S.L., Miller K.B. (2008). Optimal protein intake in the elderly. Clin. Nutr..

[6] Guller I., Russell A.P. (2010). MicroRNAs in skeletal muscle: Their role and regulation in development, disease and function. J. Physiol..

[7] Chumlea W.C., Baumgartner R.N., Vellas B.P. (1991). Anthropometry and body composition in the perspective of nutritional status in the elderly. Nutrition.

[8] Kim T.N., Won J.C., Kim Y.J., Lee E.J., Kim M.K., Park M.S., Lee S.K., Kim J.M., Ko K.S., Rhee B.D. (2013). Serum adipocyte fatty acid-binding protein levels are independently associated with sarcopenic obesity. Diabetes Res. Clin. Pract..

[9] Morais J.A., Chevalier S., Gougeon R. (2006). Protein turnover and requirements in the healthy and frail elderly. J. Nutr. Health Aging.

[10] Wilson M.M., Purushothaman R., Morley J.E. (2002). Effect of liquid dietary supplements on energy intake in the elderly. Am. J. Clin. Nutr..

[11] Dawson-Hughes B. (2003). Calcium and protein in bone health. Proc. Nutr. Soc..

[12] Dawson-Hughes B. (2003). Interaction of dietary calcium and protein in bone health in humans. J. Nutr..

[13] Thorpe M.P., Jacobson E.H., Layman D.K., He X., Kris-Etherton P.M., Evans E.M. (2008). A diet high in protein, dairy, and calcium attenuates bone loss over twelve months of weight loss and maintenance relative to a conventional high-carbohydrate diet in adults. J. Nutr..

[14] Heaney R.P., Layman D.K. (2008). Amount and type of protein influences bone health. Am. J. Clin. Nutr..

[15] Hu F.B., Stampfer M.J., Manson J.E., Rimm E., Colditz G.A., Speizer F.E., Hennekens C.H., Willett W.C. (1999). Dietary protein and risk of ischemic heart disease in women. Am. J. Clin. Nutr..

[16] Obarzanek E., Velletri P.A., Cutler J.A. (1996). Dietary protein and blood pressure. Jama.

[17] Stamler J., Elliott P., Kesteloot H., Nichols R., Claeys G., Dyer A.R., Stamler R. (1996). Inverse relation of dietary protein markers with blood pressure. Findings for 10,020 men and women in the INTERSALT Study. INTERSALT Cooperative Research Group. INTERnational study of SALT and blood pressure. Circulation.

[18] Stratton R.J., Ek A.C., Engfer M., Moore Z., Rigby P., Wolfe R., Elia M. (2005). Enteral nutritional support in prevention and treatment of pressure ulcers: A systematic review and meta-analysis. Ageing Res. Rev..

[19] U.S. Department of Agriculture, U.S. Department of Health and Human Services (2010). Dietary Guidelines for Americans.

[20] Trumbo P., Schlicker S., Yates A.A., Poos M., Food and Nutrition Board of the Institute of Medicine, The National Academies (2002). Dietary Reference Intakes for Energy, Carbohydrate, Fiber, Fat, Fatty Acids, Cholesterol, Protein and Amino Acids.

[21] Campbell W.W., Crim M.C., Dallal G.E., Young V.R., Evans W.J. (1994). Increased protein requirements in elderly people: New data and retrospective reassessments. Am. J. Clin. Nutr..

[22] Volpi E., Campbell W.W., Dwyer J.T., Johnson M.A., Jensen G.L., Morley J.E., Wolfe R.R. (2013). Is the optimal level of protein intake for older adults greater than the recommended dietary allowance?. J. Gerontol. A Biol. Sci. Med. Sci..

[23] Millward D.J., Layman D.K., Tome D., Schaafsma G. (2008). Protein quality assessment: Impact of expanding understanding of protein and amino acid needs for optimal health. Am. J. Clin. Nutr..

[24] Anthony J.C., Anthony T.G., Kimball S.R., Vary T.C., Jefferson L.S. (2000). Orally administered leucine stimulates protein synthesis in skeletal muscle of postabsorptive rats in association with increased eIF4F formation. J. Nutr..

[25] Anthony J.C., Yoshizawa F., Anthony T.G., Vary T.C., Jefferson L.S., Kimball S.R. (2000). Leucine stimulates translation initiation in skeletal muscle of postabsorptive rats via a rapamycin-sensitive pathway. J. Nutr..

[26] Gordon B.S., Kelleher A.R., Kimball S.R. (2013). Regulation of muscle protein synthesis and the effects of catabolic states. Int. J. Biochem. Cell Biol..

[27] Wolfe R.R. (2002). Regulation of muscle protein by amino acids. J. Nutr..

[28] Borsheim E., Tipton K.D., Wolf S.E., Wolfe R.R. (2002). Essential amino acids and muscle protein recovery from resistance exercise. Am. J. Physiol. Endocrinol. Metab..

[29] Mitchell C.J., Della Gatta P.A., Petersen A.C., Cameron-Smith D., Markworth J.F. (2015). Soy protein ingestion results in less prolonged p70S6 kinase phosphorylation compared to whey protein after resistance exercise in older men. J. Int. Soc. Sports Nutr..

[30] Phillips S.M., Tang J.E., Moore D.R. (2009). The role of milk- and soy-based protein in support of muscle protein synthesis and muscle protein accretion in young and elderly persons. J. Am. Coll. Nutr..

[31] Tang J.E., Moore D.R., Kujbida G.W., Tarnopolsky M.A., Phillips S.M. (2009). Ingestion of whey hydrolysate, casein, or soy protein isolate: Effects on mixed muscle protein synthesis at rest and following resistance exercise in young men. J. Appl. Physiol. 1985.

[32] Houston D.K., Nicklas B.J., Ding J., Harris T.B., Tylavsky F.A., Newman A.B., Lee J.S., Sahyoun N.R., Visser M., Kritchevsky S.B. (2008). Dietary protein intake is associated with lean mass change in older, community-dwelling adults: The Health, Aging, and Body Composition (Health ABC) Study. Am. J. Clin. Nutr..

[33] Tieland M., van de Rest O., Dirks M.L., van der Zwaluw N., Mensink M., van Loon L.J., de Groot L.C. (2012). Protein supplementation improves physical performance in frail elderly people: A randomized, double-blind, placebo-controlled trial. J. Am. Med. Dir. Assoc..

[34] Tieland M., Dirks M.L., van der Zwaluw N., Verdijk L.B., van de Rest O., de Groot L.C., van Loon L.J. (2012). Protein supplementation increases muscle mass gain during prolonged resistance-type exercise training in frail elderly people: A randomized, double-blind, placebo-controlled trial. J. Am. Med. Dir. Assoc..

[35] Wolfe R.R. (2013). Perspective: Optimal protein intake in the elderly. J. Am. Med. Dir. Assoc..

[36] Boirie Y., Dangin M., Gachon P., Vasson M.P., Maubois J.L., Beaufrere B. (1997). Slow and fast dietary proteins differently modulate postprandial protein accretion. Proc. Natl. Acad. Sci. USA.

[37] Dangin M., Boirie Y., Garcia-Rodenas C., Gachon P., Fauquant J., Callier P., Ballevre O., Beaufrere B. (2001). The digestion rate of protein is an independent regulating factor of postprandial protein retention. Am. J. Physiol. Endocrinol. Metab..

[38] Dangin M., Boirie Y., Guillet C., Beaufrere B. (2002). Influence of the protein digestion rate on protein turnover in young and elderly subjects. J. Nutr..

[39] Pennings B., Boirie Y., Senden J.M., Gijsen A.P., Kuipers H., van Loon L.J. (2011). Whey protein stimulates postprandial muscle protein accretion more effectively than do casein and casein hydrolysate in older men. Am. J. Clin. Nutr..

[40] Boirie Y., Gachon P., Beaufrere B. (1997). Splanchnic and whole-body leucine kinetics in young and elderly men. Am. J. Clin. Nutr..

[41] Paddon-Jones D., Sheffield-Moore M., Katsanos C.S., Zhang X.J., Wolfe R.R. (2006). Differential stimulation of muscle protein synthesis in elderly humans following isocaloric ingestion of amino acids or whey protein. Exp. Gerontol..

[42] Pennings B., Groen B.B., van Dijk J.W., de Lange A., Kiskini A., Kuklinski M., Senden J.M., van Loon L.J. (2013). Minced beef is more rapidly digested and absorbed than beef steak, resulting in greater postprandial protein retention in older men. Am. J. Clin. Nutr..

[43] Layman D.K., Baum J.I. (2004). Dietary protein impact on glycemic control during weight loss. J. Nutr..

[44] Layman D.K. (2003). The role of leucine in weight loss diets and glucose homeostasis. J. Nutr..

[45] Layman D.K. (2002). Role of leucine in protein metabolism during exercise and recovery. Can. J. Appl. Physiol..

[46] Katsanos C.S., Kobayashi H., Sheffield-Moore M., Aarsland A., Wolfe R.R. (2006). A high proportion of leucine is required for optimal stimulation of the rate of muscle protein synthesis by essential amino acids in the elderly. Am. J. Physiol. Endocrinol. Metab..

[47] Anthony J.C., Reiter A.K., Anthony T.G., Crozier S.J., Lang C.H., MacLean D.A., Kimball S.R., Jefferson L.S. (2002). Orally administered leucine enhances protein synthesis in skeletal muscle of diabetic rats in the absence of increases in 4E-BP1 or S6K1 phosphorylation. Diabetes.

[48] Norton L.E., Layman D.K., Bunpo P., Anthony T.G., Brana D.V., Garlick P.J. (2009). The leucine content of a complete meal directs peak activation but not duration of skeletal muscle protein synthesis and mammalian target of rapamycin signaling in rats. J. Nutr..

[49] Layman D.K., Boileau R.A., Erickson D.J., Painter J.E., Shiue H., Sather C., Christou D.D. (2003). A reduced ratio of dietary carbohydrate to protein improves body composition and blood lipid profiles during weight loss in adult women. J. Nutr..

[50] Layman D.K., Shiue H., Sather C., Erickson D.J., Baum J. (2003). Increased dietary protein modifies glucose and insulin homeostasis in adult women during weight loss. J. Nutr..

[51] Layman D.K., Evans E.M., Erickson D., Seyler J., Weber J., Bagshaw D., Griel A., Psota T., Kris-Etherton P. (2009). A moderate-protein diet produces sustained weight loss and long-term changes in body composition and blood lipids in obese adults. J. Nutr..

[52] Moore D.R., Churchward-Venne T.A., Witard O., Breen L., Burd N.A., Tipton K.D., Phillips S.M. (2015). Protein ingestion to stimulate myofibrillar protein synthesis requires greater relative protein intakes in healthy older *versus* younger men. J. Gerontol. A Biol. Sci. Med. Sci..

[53] Fulgoni V.L. (2008). Current protein intake in America: Analysis of the National Health and Nutrition Examination Survey, 2003–2004. Am. J. Clin. Nutr..

[54] Symons T.B., Sheffield-Moore M., Wolfe R.R., Paddon-Jones D. (2009). A moderate serving of high-quality protein maximally stimulates skeletal muscle protein synthesis in young and elderly subjects. J. Am. Diet. Assoc..

[55] Mamerow M.M., Mettler J.A., English K.L., Casperson S.L., Arentson-Lantz E., Sheffield-Moore M., Layman D.K., Paddon-Jones D. (2014). Dietary protein distribution positively influences 24-h muscle protein synthesis in healthy adults. J. Nutr..

[56] Murphy C.H., Churchward-Venne T.A., Mitchell C.J., Kolar N.M., Kassis A., Karagounis L.G., Burke L.M., Hawley J.A., Phillips S.M. (2015). Hypoenergetic diet-induced reductions in myofibrillar protein synthesis are restored with resistance training and balanced daily protein ingestion in older men. Am. J. Physiol. Endocrinol. Metab..

[57] Kim I.Y., Schutzler S., Schrader A., Spencer H., Kortebein P., Deutz N.E., Wolfe R.R., Ferrando A.A. (2015). Quantity of dietary protein intake, but not pattern of intake, affects net protein balance primarily through differences in protein synthesis in older adults. Am. J. Physiol. Endocrinol. Metab..

[58] Arnal M.A., Mosoni L., Boirie Y., Houlier M.L., Morin L., Verdier E., Ritz P., Antoine J.M., Prugnaud J., Beaufrere B. (1999). Protein pulse feeding improves protein retention in elderly women. Am. J. Clin. Nutr..

[59] Bouillanne O., Neveux N., Nicolis I., Curis E., Cynober L., Aussel C. (2014). Long-lasting improved amino acid bioavailability associated with protein pulse feeding in hospitalized elderly patients: A randomized controlled trial. Nutrition.

[60] Bouillanne O., Curis E., Hamon-Vilcot B., Nicolis I., Chretien P., Schauer N., Vincent J.P., Cynober L., Aussel C. (2013). Impact of protein pulse feeding on lean mass in malnourished and at-risk hospitalized elderly patients: A randomized controlled trial. Clin. Nutr..

[61] Wall B.T., Hamer H.M., de Lange A., Kiskini A., Groen B.B., Senden J.M., Gijsen A.P., Verdijk L.B., van Loon L.J. (2013). Leucine co-ingestion improves post-prandial muscle protein accretion in elderly men. Clin. Nutr..

[62] Casperson S.L., Sheffield-Moore M., Hewlings S.J., Paddon-Jones D. (2012). Leucine supplementation chronically improves muscle protein synthesis in older adults consuming the RDA for protein. Clin. Nutr..

[63] Rasmussen B.B., Wolfe R.R., Volpi E. (2002). Oral and intravenously administered amino acids produce similar effects on muscle protein synthesis in the elderly. J. Nutr. Health Aging.

[64] Cuthbertson D., Smith K., Babraj J., Leese G., Waddell T., Atherton P., Wackerhage H., Taylor P.M., Rennie M.J. (2005). Anabolic signaling deficits underlie amino acid resistance of wasting, aging muscle. FASEB J..

[65] Sakuma K., Aoi W., Yamaguchi A. (2015). Current understanding of sarcopenia: Possible candidates modulating muscle mass. Pflugers Arch..

[66] Sakuma K., Aoi W., Yamaguchi A. (2014). The intriguing regulators of muscle mass in sarcopenia and muscular dystrophy. Front. Aging Neurosci..

[67] Kimball S.R., Jefferson L.S. (2006). Signaling pathways and molecular mechanisms through which branched-chain amino acids mediate translational control of protein synthesis. J. Nutr..

[68] Kimball S.R., Jefferson L.S. (2005). Role of amino acids in the translational control of protein synthesis in mammals. Semin. Cell Dev. Biol..

[69] Kimball S.R., Jefferson L.S. (2004). Regulation of global and specific mRNA translation by oral administration of branched-chain amino acids. Biochem. Biophys. Res. Commun..

[70] Wilkinson D.J., Hossain T., Hill D.S., Phillips B.E., Crossland H., Williams J., Loughna P., Churchward-Venne T.A., Breen L., Phillips S.M. (2013). Effects of leucine and its metabolite β-hydroxy-β-methylbutyrate on human skeletal muscle protein metabolism. J. Physiol..

[71] Katsanos C.S., Kobayashi H., Sheffield-Moore M., Aarsland A., Wolfe R.R. (2005). Aging is associated with diminished accretion of muscle proteins after the ingestion of a small bolus of essential amino acids. Am. J. Clin. Nutr..

[72] Paddon-Jones D., Sheffield-Moore M., Creson D.L., Sanford A.P., Wolf S.E., Wolfe R.R., Ferrando A.A. (2003). Hypercortisolemia alters muscle protein anabolism following ingestion of essential amino acids. Am. J. Physiol. Endocrinol. Metab..

[73] Guillet C., Prod’homme M., Balage M., Gachon P., Giraudet C., Morin L., Grizard J., Boirie Y. (2004). Impaired anabolic response of muscle protein synthesis is associated with S6K1 dysregulation in elderly humans. FASEB J..

[74] Fry C.S., Drummond M.J., Glynn E.L., Dickinson J.M., Gundermann D.M., Timmerman K.L., Walker D.K., Volpi E., Rasmussen B.B. (2013). Skeletal muscle autophagy and protein breakdown following resistance exercise are similar in younger and older adults. J. Gerontol. A Biol. Sci. Med. Sci..

[75] Drummond M.J., Miyazaki M., Dreyer H.C., Pennings B., Dhanani S., Volpi E., Esser K.A., Rasmussen B.B. (2009). Expression of growth-related genes in young and older human skeletal muscle following an acute stimulation of protein synthesis. J. Appl. Physiol. 1985.

[76] Drummond M.J., Dickinson J.M., Fry C.S., Walker D.K., Gundermann D.M., Reidy P.T., Timmerman K.L., Markofski M.M., Paddon-Jones D., Rasmussen B.B. (2012). Bed rest impairs skeletal muscle amino acid transporter expression, mTORC1 signaling, and protein synthesis in response to essential amino acids in older adults. Am. J. Physiol. Endocrinol. Metab..

[77] Kelijman M. (1991). Age-related alterations of the growth hormone/insulin-like-growth-factor I axis. J. Am. Geriatr. Soc..

[78] Ceda G.P., Dall’Aglio E., Maggio M., Lauretani F., Bandinelli S., Falzoi C., Grimaldi W., Ceresini G., Corradi F., Ferrucci L. (2005). Clinical implications of the reduced activity of the GH-IGF-I axis in older men. J. Endocrinol. Investig..

[79] Schurch M.A., Rizzoli R., Slosman D., Vadas L., Vergnaud P., Bonjour J.P. (1998). Protein supplements increase serum insulin-like growth factor-I levels and attenuate proximal femur bone loss in patients with recent hip fracture. A randomized, double-blind, placebo-controlled trial. Ann. Intern. Med..

[80] Morley J.E. (2010). Anorexia, weight loss, and frailty. J. Am. Med. Dir. Assoc..

[81] Locher J.L., Ritchie C.S., Roth D.L., Sen B., Vickers K.S., Vailas L.I. (2009). Food choice among homebound older adults: Motivations and perceived barriers. J. Nutr. Health Aging.

[82] Wolfe R.R. (2006). The underappreciated role of muscle in health and disease. Am. J. Clin. Nutr..

[83] Keller H.H. (1993). Malnutrition in institutionalized elderly: How and why?. J. Am. Geriatr. Soc..

[84] Morley J.E. (1997). Anorexia of aging: Physiologic and pathologic. Am. J. Clin. Nutr..

[85] Paddon-Jones D., Sheffield-Moore M., Zhang X.J., Volpi E., Wolf S.E., Aarsland A., Ferrando A.A., Wolfe R.R. (2004). Amino acid ingestion improves muscle protein synthesis in the young and elderly. Am. J. Physiol. Endocrinol. Metab..

[86] Volpi E., Ferrando A.A., Yeckel C.W., Tipton K.D., Wolfe R.R. (1998). Exogenous amino acids stimulate net muscle protein synthesis in the elderly. J. Clin. Investig..

[87] Demling R.H., DeSanti L. (1998). Increased protein intake during the recovery phase after severe burns increases body weight gain and muscle function. J. Burn Care Rehabil..

[88] Hughes M.S., Kazmier P., Burd T.A., Anglen J., Stoker A.M., Kuroki K., Carson W.L., Cook J.L. (2006). Enhanced fracture and soft-tissue healing by means of anabolic dietary supplementation. J. Bone Jt. Surg. Am..

